# BF_3_-OEt_2_ Catalyzed C3-Alkylation of Indole: Synthesis of Indolylsuccinimidesand Their Cytotoxicity Studies

**DOI:** 10.3390/molecules26082202

**Published:** 2021-04-11

**Authors:** Iqbal N. Shaikh, Abdul Rahim, Shaikh Faazil, Syed Farooq Adil, Mohamed E. Assal, Mohammad Rafe Hatshan

**Affiliations:** 1 Department of Chemistry, Poona College of Arts, Science and Commerce, Camp, Pune 411 001, India; shaikhiqbaln@gmail.com (I.N.S.); abdulrahimakot@gmail.com (A.R.); 2 Department of Chemistry, College of Science, King Saud University, P.O. Box 2455, Riyadh 11451, Saudi Arabia; meassal@ksu.edu.sa (M.E.A.); mhatshan@ksu.edu.sa (M.R.H.)

**Keywords:** C3-alkylation, indole, indolylsuccinimides, cytotoxicity, anticancer

## Abstract

A simple and efficient BF_3_-OEt_2_ promoted C3-alkylation of indole has been developed to obtain3-indolylsuccinimidesfrom commercially available indoles and maleimides, with excellent yields under mild reaction conditions. Furthermore, anti-proliferative activity of these conjugates was evaluated against HT-29 (Colorectal), Hepg2 (Liver) and A549 (Lung) human cancer cell lines. One of the compounds, **3w**, having *N*,*N*-Dimethylatedindolylsuccinimide is a potent congener amongst the series with IC_50_ value 0.02 µM and 0.8 µM against HT-29 and Hepg2 cell lines, respectively, and compound **3i** was most active amongst the series with IC_50_ value 1.5 µM against A549 cells. Molecular docking study and mechanism of reaction have briefly beendiscussed. This method is better than previous reports in view of yield and substrate scope including electron deficient indoles.

## 1. Introduction

**Figure 1:**
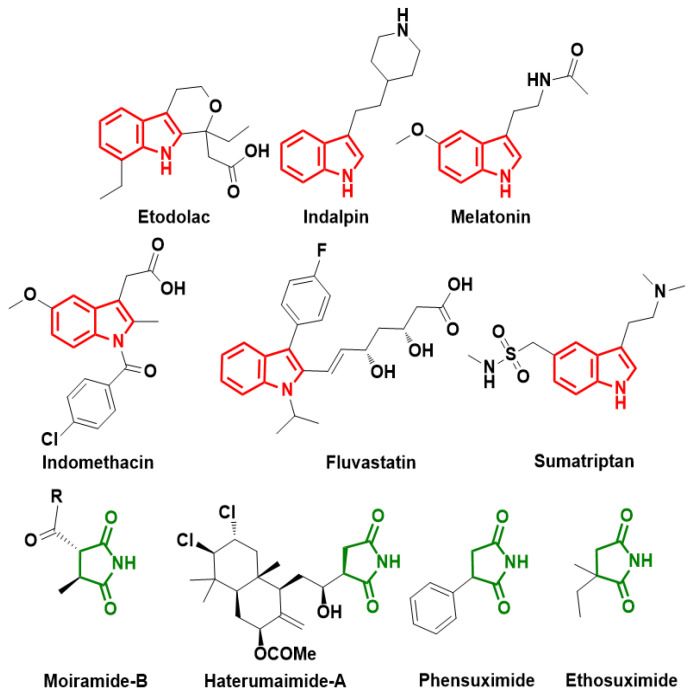
Indole and succinimide rings present in natural products and drug candidates.

The properties like anticancer [1,2], antioxidant [3,4,5], antirheumatoidal [6,7] and anti-HIV [8,9,10] has made indole a privileged scaffold and its derivatives such as indolylsuccinimide are important intermediates in organic synthesis and pharmaceuticals [11,12,13,14,15,16,17,18]. The indole ring system is present in many commercially marketed drugs (Figure 1) [19,20,21]. Moreover, different indole derivatives targeting regulation of PI3K/Akt/mTOR/NF-κB and other signaling pathways have been reported [22,23,24]. Maleimide and its *N*-substituted derivatives are potent and selective telomerase inhibitors suitable for cancer therapy [25]. This moiety is used as a bridge to the disulfide bond present in protein and protein PEGylation [26,27] and as a linker in antibody-drug conjugates to increase their tolerability, intra-tumoral drug delivery as well as to improve the therapeutic efficacy [28,29,30]. Similarly, Ru(II) and Pt(IV) based compounds with maleimide functionality have been prepared to selectively deliver these compounds to the tumor [31,32]. Additionally, maleimide-derived molecule MIRA-1 reactivates mutant p53 in living cells and induces mutant p53-dependent cell death in different human tumor cells [33,34].

On the other hand, another interesting molecule, the succinimide which is found in many natural products, is also noticed in several clinical drug candidates, indicating that this scaffold plays a vital role in exhibiting a wide range of biological and pharmaceutical activities (Figure 1) [35,36,37,38]. Further, succinimides can be easily reduced into 5-membered pyrrolidine rings, γ-lactams and lactims, which by themselves are useful natural product motifs and can be oxidized to maleimides [39,40].

Thus, importance of developing methods to synthesize indolyl-derivatives can never be overestimated, which is also indicated by the large number of reports appearing consistently in various journals from the past decades [41,42,43,44,45,46]. Most importantly, their synthesis with selective functionalization has become an active research area [47,48,49]. In contrast to the conventional approaches, the direct functionalization of indole has emerged more recently as a preferred methodology as it is more practical and reduces the number of steps [50,51,52,53,54]. Among the several approaches for the direct substitution of indoles, transition metal catalyzed C-H activations are more attractive [55,56,57,58]. However, positions C2 and C3 are the most activated ones, thus direct C3 alkylations are still limited. Therefore, allylic alkylations [59,60,61,62,63,64,65], Baylis–Hillman [66,67,68,69] and more frequently Friedel-Craft type of reactions [70,71,72,73,74,75] are especially useful to achieve this transformation.

Apart from the various indolyl-derivatives synthesized, the preparation of indolylsuccinimidesneeds to be paid attention to as the previously reported methods have several drawbacks, such as the general method of preparation, which is carried out by the reaction of indoles with maleimides by refluxing in acetic acid. However, this reaction requires long reaction time (up to 3–5 days) and the products are obtained in low yields even after use of excess amounts of reactants [76,77]. In case of acid-catalyzed conjugate addition of indoles, careful control of the acidity to prevent side reactions such as dimerization or polymerization is required [78]. Other methods involve the use of Pd and Ru metals for cross-coupling between indole and dihalomaleimides for the synthesis of 3-indolylsuccinimides and 2-indolylsuccinimides, respectively [79,80,81,82]. To the best of our knowledge, only two reactions were reported previously which involves the conjugate addition of indoles to maleimide compounds in the presence of Lewis acids such as ZnCl_2_/AlCl_3_ (1 mmol) [76,77,78]. However, these reactions were performed in DCE or nitromethane under heating or refluxing condition using electron rich indoles. It was observed that the electron-deficient indoles provide unsatisfactory yield and more often very low reaction yield. Furthermore, there are many methods to functionalize the C3 position of the indole nucleus but very few of them allow the use of free N-H indole skeletons.

**Scheme 1:**
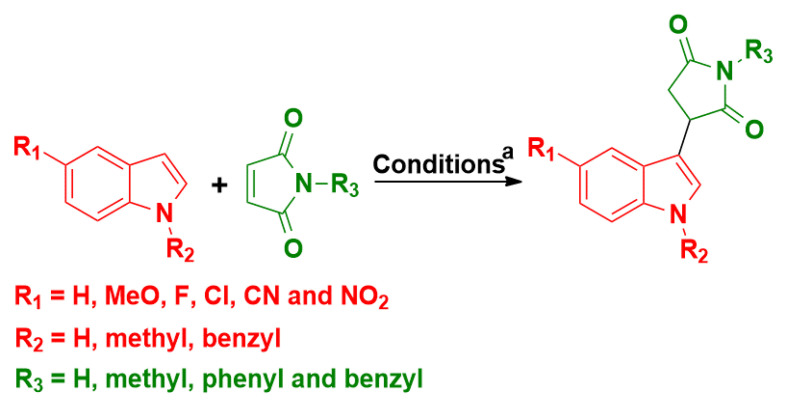
Synthesis of indolylsuccinimides; ^a^ Reagents and conditions: BF_3_OEt_2_ (50 mol%), ethyl acetate, 60 °C, 6 h.

Thus, an efficient, economical and environmentally benign commercially available or new catalyst is highly desirable for this procedure. In continuation of our work with regard to the preparation of new catalyst for various organic transformations, [79,80,81] we herein report a strategy in which commercially available boron trifluoride diethyl etherfunctions as an effective catalyst [82,83] for the synthesis of indolylsuccinimides (Scheme 1). Interestingly, unlike earlier procedures, it offers a convenient single step reaction and works well with the electron-deficient indoles under mild reaction conditions. These indolylsuccinimide conjugates were evaluated for their cytotoxicity against HT-29, Hepg2 and A549 human cancer cell lines and moreover molecular docking studies were also carried out to understand their binding mode to the receptor.

## 2. Results and Discussion

### 2.1. Chemistry

**Scheme 2:**
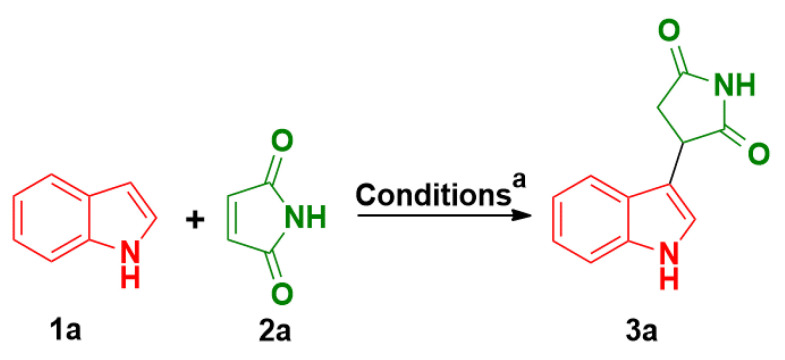
Optimization of reaction.

Initially, 1*H*-indole (**1**) (Scheme 2) is taken as a model substrate to study this reaction, because the product, 3-indolylsuccinimide **3a** is a solidandcan be easily purified. As shown in Table 1, the reaction between 1*H*-indole (**1a**) and maleimide (**2a**) does not take place at room temperature in the absence of any catalyst up to 12 h (Table 1, entry 1). However, use of iodine in ethanol solvent furnished the desired compound with very low yield (Table 1, entry 2). Increasing the temperature to 60 °C did not show any remarkable change in the yield. Switching the solvent from ethanol to methanol and acetonitrile slightly increased the yield (Table 1, entries 3 and 4). Lewis acid catalyzed addition of indole to maleimide has been previously reported employingZnCl_2_, however this catalyst is found to be inefficient as it also provided very low yield for the reaction under the aforesaid conditions (Table 1, entries 5–7). Moreover, attempts using copper and silver catalyst also failed to give the desired products in this reaction (Table 1, entries 8–12). Similarly, the use of cerium ammonium nitrate (CAN) and boric acid as catalysts too did not yield the required products (Table 1, entries 13 and 14). 

Later, the use of classic Lewis acid boron trifluoride ethyl ether (BF_3_OEt_2_) as catalyst is attempted and found to be efficient as the desired product is obtained in relatively good yield (Table 1, entry 15) when compared to the other reactions attempted. Moreover, increasing the reaction temperature from room temperatureto 60 °C in acetonitrile as solvent displayed a nearly two-fold increases in the yield (Table 1, entry 16). However, decrease in yield is reported when solvent is changed from acetonitrile to methyl alcohol (entry 17). Further, change in solvent from protic to aprotic viz. 1,2-dichloroethane (entry 18) increased in the product yield is observedA substantial yield i.e., ~78%, of 3-indolylsuccinimide **3a** is obtained when ethyl acetate is taken as solvent and the reaction time reduced significantly from 12 h to 6 h (Table 1, entry 19). Thus, the optimal reaction conditions for the efficient synthesis of 3-indolylsuccinimide **3a**, catalyzed by BF_3_OEt_2_ (0.5 mmol) requires **1a** (1.0 mmol) and **2a** (1.0 mmol) wherein ethyl acetate will be used as a solvent at 60 °C upon stirring for 6 h, hence this condition will be extended to the synthesis of a variety of Indolylsuccinimides to afford the desired product in 78% yield.

**Scheme 3:**
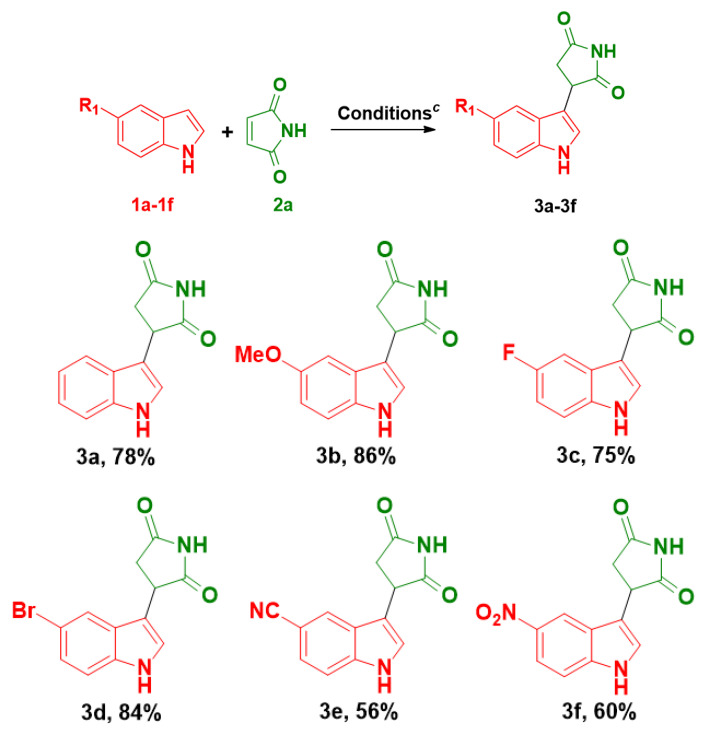
Substrate scope of indoles; *^c^* Reaction conditions: **1a-1f** (1.0 mmol), **2a** (1.0 mmol), BF_3_OEt_2_ (0.5 mmol), heated in 5 mL of solvent within 6 h.

With the optimized reaction conditions established, the substrate scope of BF_3_OEt_2_-catalysed C3-alkylation reaction with different indole substrates is investigated. A series of 5-substituted indoles are found to undergo the desired coupling to give the corresponding products in moderate to excellent yield (56−86%, Scheme 3). The structure of resultant compounds is established on the basis of their ^1^H, ^13^C NMR and HRMS spectra. However, some of the products were reported previously are corroborated with their reported data and found in accordance (Appendix A.) [76,77,84].

**Scheme 4:**
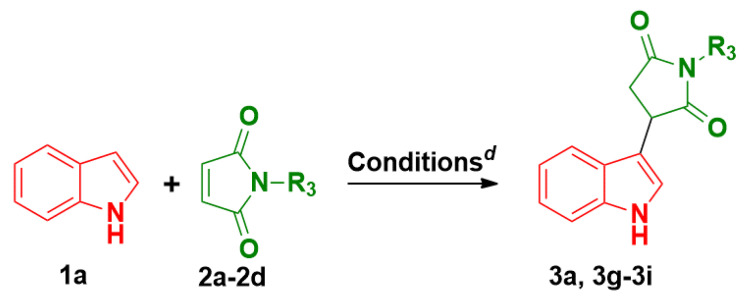

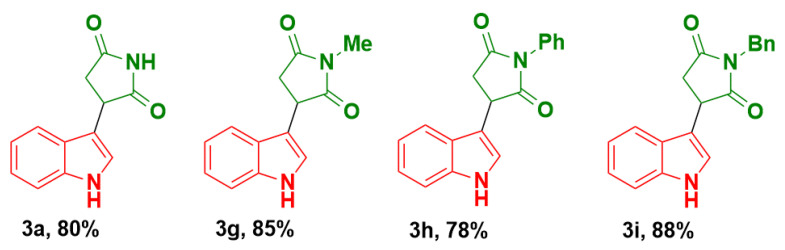
Substrate scope of maleimides; *^d^* Reaction conditions: **1a** (1.0 mmol), **2a-2d** (1.0 mmol), BF_3_OEt_2_ (0.5 mmol), heated in 5 mL of solvent within 6 h.

The electronic nature of the indoles was shown to have more influence on the reaction efficiency. The presence of electron-donating groups (5-OMe) significantly increased the yield as it is obvious in case of electrophilic substitution reaction of indole that provides 86% of **3b**. However, electron-deficient groups (5-CN and 5-NO_2_) had drastic effect on reactivity, and the corresponding 3-indolylsuccinimides were obtained in relatively low yields (56% and 60% for **3e** and **3f**) than the electron-neutral group containing compound **3a** with 78% yield. The time required for the completion of this reaction is found between 2and 6 h. The optimal reaction conditions are also compatible with halogenated indole (i.e., 5-F and 5Br), with the corresponding products **3c** and **3d** obtained in 75% and 84% yields (Scheme 3). We next investigated the scope of *N*-alkylated maleimides as substrates and the results are displayed in Scheme 4. All the *N*-alkylated maleimides reacted well, giving the desired products **3g**–**3i** in 78–88% yield. Notably, *N*-methyl and *N*-benzyl maleimides exhibited excellent reactivity than *N*-Phenyl (**3h**, 78%) to give corresponding products **3g** and **3i** in 83% and 88% yield. These results indicate that *N*-protected maleimides havesome effect on the reaction.

**Scheme 5:**
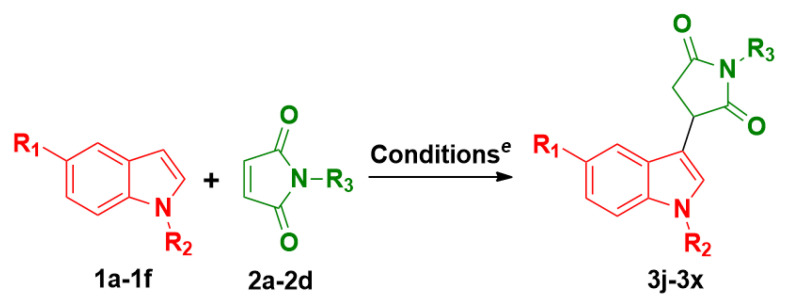

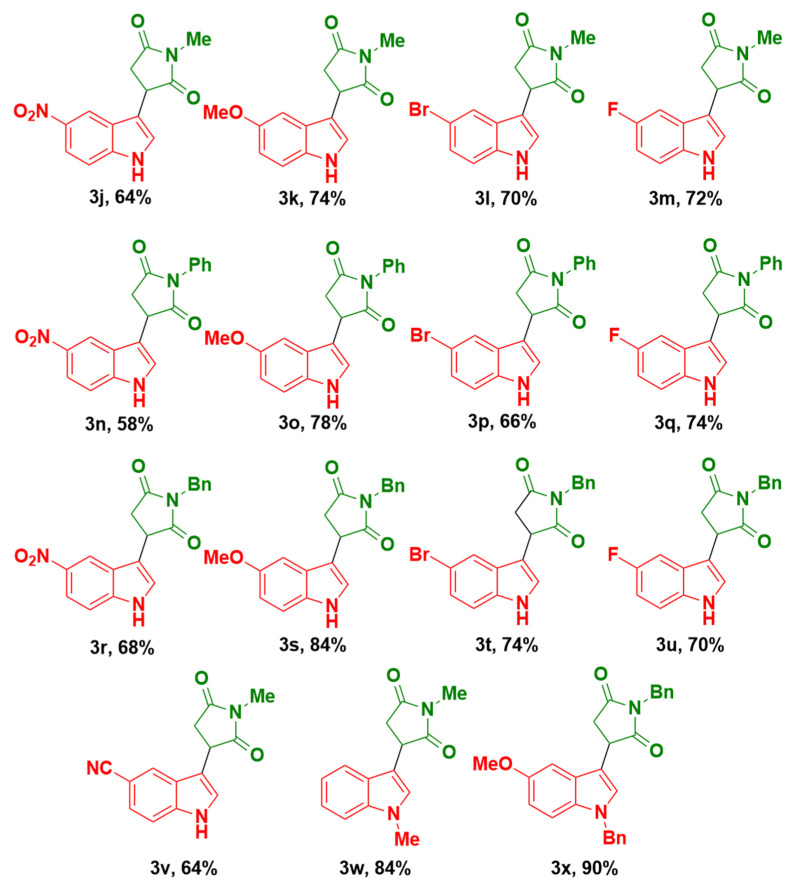
Scope of indole and maleimides; *^e^* Reaction conditions: **1a-1f** (1.0 mmol), **2a-2d** (1.0 mmol), BF_3_OEt_2_ (0.5 mmol), heated in 5 mL of solvent within 6 h.

To extend the scope, we also made substituted indoles products (**3j-3x**) with good to excellent yield (58–90%). Electron-deficient groups (5-CN and 5-NO_2_) containing compounds were formed in relatively low yield (**3j**, **3n**, **3r** and **3v** with 64%, 58%, 68% and 64%) than electron donating group (MeO, **3k**, 74%) and halogenated compounds (Br and Cl, **3l-3m**, 70% and 72%). Moreover, the reaction of *N*-benzyl maleimides furnished the products in excellent yield compared to their *N*-methyl and *N*-phenyl counterparts. Interestingly, when *N*-alkylated indole and *N*-alkylatedmaleimide was investigated, the yield of the products can rise drastically (**3w-3x**, 84% and 90%) as shown in Scheme 5.

#### Plausible Mechanism

**Scheme 6:**
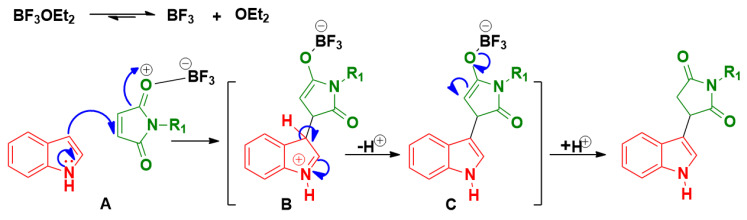
Plausible mechanism.

BF_3_-etherate catalyst serves as a source of boron trifluoride via the equilibrium [85,86] shown below. The BF_3_ coordinate with the oxygen of maleimide, inducing reactions of the resulting adducts A with indole nucleophile to generate intermediate B which on deprotonationconverted to intermediate C hence restoration of aromaticity. Finally, this gives rise to the desire product Scheme 6.

### 2.2. Biological Evaluation

To access the cytotoxicity, all compounds were screened against three human cancer cell lines namely HT-29, Hepg2 and A549 by MTT assay [87,88]. The compound **3w** was found to be the most potent congener amongst the series with IC_50_ value 0.02 µM and 0.8 µM against HT-29 and Hepg2 cell lines, respectively and compound **3i** was most active amongst the series with IC_50_ value 1.5 µM against A549 cells.

#### 2.2.1. Structure Activity Relationship 

Indole based bioactive molecules are reported in literature for diversified activity including anti-canceractivity [89].

It is observed from the Table 2 that compounds having unprotected indole and succinimide moieties (**3a–3f**) showed favorable activity against HT-29 and Hepg-2 cell lines with IC_50_ values ranging from 3.6 to 9.1 µM. Interestingly, compound **3b** has IC_50_ value of 3.6 µM against Hepg-2 cells. However, *N*-methylated, *N*-phenyland*N*-benzylatedsuccinimids with unprotected indoles were relatively less active against these cell lines. Moreover, *N*-methylated and *N*-benzylatedsuccinimides (**3i, 3k-3m, 3u** and **3v**) are more active against A549 cells than *N*-phenyl compounds with IC_50_ ranging from 1.5 to 8.7 µM. Remarkably, compound **3w** having *N*-methyl on both indole as well as succinimide rings showed potential cytotoxicity with IC_50_ values of 0.02 and 0.8 µM against HT-29 and Hepg-2 cell lines, respectively. In view of electron rich and deficient indoles no remarkable distintion was observed, however these indoles were more active against HT-29 and Hepg-2 cells than A549 cells. Interestingly, halogenated indoles showed enhanced effect against A549 cells.

#### 2.2.2. Molecular Docking Studies

Molecular docking studies were carried out with a view to understand the site of binding by these compounds. It is evident from the literature that various indolylmaleimidederivatives show cytotoxic properties by inhibiting the cyclin-dependent kinases and therefore molecular docking studies were performed at the ATP binding pocket of CDK2 as a model kinase [90]. A number of structural studies have demonstrated that most of inhibitors bind to CDK2 in a fashion similar to Staurosporine (which is a known kinase inhibitor), the adenine ring of ATP, forming a triplet of hydrogen bonds to the peptide backbone of residues Glu81 and Leu83, which resides in the hydrophilic hinge region at the back of the binding pocket [91]. The crystal structure of Staurosporine in complex with CDK2 provides insight into the interactions responsible for high-affinity binding to a variety of kinases. The crystal structure of the protein was obtained from Protein Data Bank (PDB ID 1AQ1) [31], necessary corrections to the protein were carried out using Protein Preparation Wizard from the Schrodinger package and 3D structures were generated by Schrödinger suite (Schrödinger’sLigPrep program). Molecular docking studies were performed by using a GLIDE docking module of Schrödinger suite and the results were analyzed on the basis of the GLIDE docking score and molecular recognition interactions. All the 3D figures were obtained using Schrödinger Suite 2014-3 [92].

**Figure 2:**
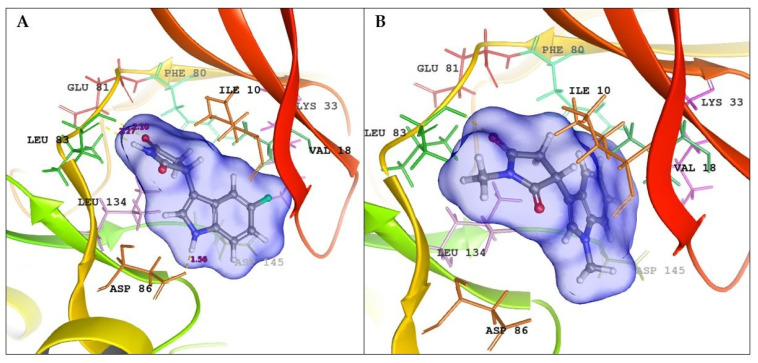
(**A**) Binding pose of compound **3c** in ATP binding pocket of CDK2. (**B**) Binding pose of compound **3w** in ATP binding pocket of CDK2. Compounds **3c** and **3w** shown in stick and colored by the atom type (carbon: grey; oxygen: red; nitrogen: blue; fluorine: green).

Docking studies were performed on **3c** and **3w**, which suggests a reasonable binding mode in the ATP-binding pocket of CDK2 (Figure 2). This binding mode is very similar to the Staurosporine binding mode. Likewise, compound **3c** hydrogen bond to the backbone carbonyl of Glu81 and to the backbone amine of Leu83 are formed, compound **3w** formed hydrogen bond with leu83. The indole ring is located in the hydrophobic cleft formed by the amino acids Ile10, Val18, Lys33, Phe80, Leu134, and Asp145.

## 3. Materials and Methods

### 3.1. Preparation of Compounds

The detailed procedure of the synthesis is given in Appendix A section.

### 3.2. Biological Activity

The detailed procedure employed for the biological activity is given in Appendix A section.

## 4. Conclusions

A simple and efficient BF_3_-OEt_2_ promoted C3-alkylation of indoles has been developed to access 3-indolylsuccinimidesfrom commercially available indoles and maleimides, in good to excellent yields (64–90%) (under mild reaction conditions. This is an improved protocol compared to the previously reported ones, in view of yield and substrate scope including electron-deficient indoles. Furthermore, anti-proliferative activity of these congeners was evaluated against HT-29, Hepg2 and A549 human cancer cell lines. Compound **3w** was found to be the most potent amongst the series with IC_50_ value of 0.02 µM and 0.8 µM against HT-29 and Hepg2 cell lines, respectively and compound **3i** was most active amongst the series with IC_50_ value 1.5 µM against A549 cell line.

## Figures and Tables

**Table 1:** Optimization of the reaction conditions *^a^*.

Entry	Catalyst	Solvent *	Temp (°C)	Time (h)	Yield(%) *^b^*
1	-	DCE	r.t/60 *^c^*	12	0
2	I_2_	EtOH	r.t/60	12	5
3	I_2_	MeOH	r.t/60	12	18
4	I_2_	MeCN	r.t/60	12	25
5	ZnCl_2_	MeCN	r.t/60	12	trace
6	ZnCl_2_	DCE	r.t/60	12	10
7	ZnCl_2_	EtOAc	r.t/60	12	trace
8	Cu(OAc)_2_	MeCN	r.t/60	12	N.R
9	Cu(OTf)_2_	MeCN	r.t/60	12	N.R
10	Cu(NO_3_)_2_	MeCN	r.t/60	12	trace
11	AgCl	MeCN	r.t/60	12	N.R
12	AgOTf	MeCN	r.t/60	12	N.R
13	CAN	MeCN	r.t/60	12	N.R
14	H_3_BO_3_	MeCN	r.t/60	12	0
15	BF_3_OEt_2_	MeCN	r.t	12	30
16	BF_3_OEt_2_	MeCN	60	12	52
17	BF_3_OEt_2_	MeOH	60	12	44
18	BF_3_OEt_2_	DCE	60	12	62
**19**	**BF_3_OEt_2_**	**EtOAc**	**60**	**6**	**78**

*^a^* Reaction conditions: **1a** (1.0 mmol), **2a** (1.0 mmol), catalyst (0.5 mmol), heated in 5 mL of solvent within 12 h. *^b^* Products were obtained in isolated yields based on indole. *^c^* Reaction first stirred at r.t. if starting material not consumed totally then temperature shifted to 60 °C. * DCE-dichloroethane, EtOH- ethanol, MeOH-methanol, MeCN-acetonitrile, EtOAc-ethyl acetate.

**Table 2:** Cytotoxicity of indolylsuccinimide analogues.

Entry	Compound	Cancer Cell Lines (IC_50_ µ/M) *^f^*		
		HT-29 *^g^*	Hepg2 *^h^*	A549 *^i^*
1	**3a**	4.32 (±0.06)	5.2 (±0.03)	10.9 (±0.03)
2	**3b**	4.67 (±0.16)	03.6 (±0.02)	9.7 (±0.22)
3	**3c**	4.36 (±0.33)	05.8 (±0.03)	2.1 (±0.20)
4	**3d**	7.9 (±0.32)	09.1 (±0.04)	6.4 (±0.14)
5	**3e**	06.2 (±0.23)	05.6 (±0.06)	18.2 (±0.22)
6	**3f**	05.4 (±0.13)	04.9 (±0.01)	10.7 (±0.05)
7	**3g**	10.7 (±0.23)	11.4 (±0.03)	9.6 (±0.13)
8	**3h**	08.4 (±0.06)	15.9 (±0.03)	10.6 (±0.34)
9	**3i**	24.8 (±0.02)	20.3 (±0.03)	1.5 (±0.45)
10	**3j**	07.8 (±0.03)	06.9 (±0.11)	9.1 (±0.33)
11	**3k**	10.6 (±0.12)	13.0 (±0.13)	2.5 (±0.12)
12	**3l**	10.9(±0.03)	13.6 (±0.035)	3.9 (±0.34)
13	**3m**	19.9 (±0.12)	14.5 (±0.6)	3.5 (±0.01)
14	**3n**	09.1(±0.06)	7.8 (±0.21)	12.9 (±0.22)
15	**3o**	06.6(±0.4)	16.5 (±0.22)	14.4 (±0.03)
16	**3p**	12.5±0.03	18.7 (±0.20)	11.3 (±0.03)
17	**3q**	10.2 (±0.33)	5.9 (±0.05)	7.6 (±0.03)
18	**3r**	10.3 (±0.45)	12.5 (±0.07)	8.7 (±0.06)
19	**3s**	23.6 (±0.67)	19.5 (±0.56)	15.8 (±0.22)
20	**3t**	22.5 (±0.34)	18.6 (±0.05)	11.4 (±0.17)
21	**3u**	10.7 (±0.23)	16.7 (±0.67)	2.4 (±0.14)
22	**3v**	08.5 (±0.13)	07.2 (±0.09)	3.6 (±0.24)
23	**3w**	0.02 (±0.02)	0.8 (±0.05)	6.3 (±0.12)
24	**3x**	28.2 (±0.34)	17.3 (±0.22)	14.7 (±0.12)
25	Doxorubicin	01.2 (±0.03)	01.8 (±0.01)	0.9 (±0.10)

*^f^* 50% Inhibitory concentration after 48 h of compounds treatment and the values are average of three individual experiments. *^g^* Human colorectal adenocarcinoma cells, *^h^* Human liver cancer and *^i^* Human lung cancer.

## Data Availability

The data presented in this study are available on request from the corresponding author.

## Sample Availability

Samples of the compounds can be checked with the author, Faazil Shaikh (faazil.shaikh@poonacollege.edu.in).

## Appendix A. Experimental Section

### Appendix A.1. Chemistry

All reagents, starting materials, and solvents were purchased from Aldrich (Sigma-Aldrich, St. Louis, MO, USA) or Alfa Aesar (Johnson Matthey Company, Ward Hill, MA, USA) and used without further purification. Reactions were monitored by TLC, performed on silica gel glass plates containing 60 F-254, and visualization on TLC was achieved by UV light or using an iodine indicator. Column chromatography was performed with Merck 60–120 mesh silica gel. ***^1^H*** and ***^13^C* NMR** spectra were recorded with 75, 100, 300, 400, and 500 MHz spectrometer in CDCl_3_ and DMSO-d_6_ solutions. Chemical shifts (**δ**) are expressed in ppm relative to the internal standard TMS and multiplicities of NMR signals are represented as singlet (s), doublet (d), triplet (t), quartet (q), double doublet (dd), triplet of doublet (td) and multiplets (m). High-resolution mass spectra (**ESI-HRMS**) were obtained by using ESI-QTOF mass spectrometer. Melting points were determined on an Electro-thermal melting point apparatus and are uncorrected.

### Appendix A.2. General Procedure for the Synthesis of Compounds (3a–3x)

A mixture of 117 mg (1 mmol) indole, 97 mg (1 mmol) maleimide andapproximately a drop of BF_3_OEt_2_ (0.5 mmol) in 5 mL of ethyl acetate solvent was stirred at 60 °C for 6 h. Completion of reaction was monitor by TLC. After completion, the mixture was cooled to room temperature. In some cases where the product succinimide appeared as a solid from the reaction mixture filtered and recrystallized. Otherwise, H_2_O (20 mL) was added to the solution and extracted with EtOAc (2 × 25 mL). The combined organic layer was dried over anhydrous Na_2_SO_4_ and concentrated by rotary evaporation to afford crude product which was further purified by column chromatography using EtOAc and hexane as solvent system.

*3-(1H-Indole-3-yl) pyrrolidine-2,5-dione* (**3a**). White solid (80%), M.P.: 192–194 °C; ^1^H NMR (300 MHz, DMSO-d_6_) δ 11.31 (s, 1H), 11.03 (s, 1H), 7.39 (dd, *J* = 12.5, 8.1 Hz, 2H), 7.33 (s, 1H), 7.10 (t, *J* = 7.5 Hz, 1H), 6.99 (t, *J* = 7.4 Hz, 1H), 4.33 (dd, *J* = 9.4, 5.2 Hz, 1H), 3.18 (dd, *J* = 18.0, 9.5 Hz, 1H), 2.76 (dd, *J* = 18.0, 5.2 Hz, 1H); ^13^C NMR (75 MHz, CDCl_3_+ DMSO-d_6_): δ 178.9, 177.0, 136.0, 124.9, 122.0, 120.9, 118.3, 117.5, 111.1, 110.0, 38.7, 37.0; HRMS (ESI) calculated for C_12_ H_11_O_2_N_2_ [M+ H]^+^ 215.0815; found: 215.0817.

*3-(5-MetHoxy-1H-indole-3-yl) pyrrolidine-2,5-dione* (**3b**). White solid (86%), M.P.: 206–208 °C; ^1^H NMR (300 MHz, DMSO-d_6_) δ 11.08 (s, 1H), 10.27 (s, 1*H*), 7.21 (d, *J* = 8.8 Hz, 1H), 7.05 (d, *J* = 2.1 Hz, 1H), 6.82 (d, *J* = 1.9 Hz, 1H), 6.71 (dd, *J* = 8.8, 2.2 Hz, 1H), 4.18 (dd, *J* = 9.4, 5.1 Hz, 1H), 3.73 (s, 3H), 2.74 (dd, *J* = 18.2, 5.1 Hz, 1H); ^13^C NMR (75 MHz, CDCl_3_+ DMSO-d_6_) δ 178.8, 177.0, 152.9, 131.2, 125.4, 122.5, 111.7, 110.9, 109.7, 99.7, 55.0, 38.7, 37.0; HRMS (ESI) calculated for C_13_ H_13_O_3_N_2_ [M+ H]^+^ 245.0920; found: 245.0923.

*3-(5-Fluoro-1H-indole-3-yl) pyrrolidine-2,5-dione* (**3c**). White solid (75%), M.P.: 198–199 °C; ^1^H NMR (300 MHz, DMSO-d_6_) δ 11.30 (s, 1H), 11.14 (s, 1H), 7.41 (d, J = 2.3 Hz, 1H), 7.37 (dd, *J* = 8.8, 4.6 Hz, 1H), 7.19 (dd, *J* = 10.1, 2.3 Hz, 1H), 6.95 (td, *J* = 9.2, 2.5 Hz, 1H), 4.33 (dd, *J* = 9.4, 5.5 Hz, 1H), 3.17 (dd, *J* = 18.0, 9.5 Hz, 1H), 2.78 (dd, *J* = 18.0, 5.5 Hz, 1H); ^13^C NMR (75 MHz, CDCl_3_+ DMSO-d_6_) δ 179.1, 177.4, 158.6 (d), 155.5, 132.9, 125.6, 125.5, 124.1, 112.3, 112.2, 110.5, 110.0, 109.6, 102.9, 102.6, 39.0, 37.2; HRMS (ESI) calculated for C_12_H_10_FO_2_N_2_ [M+ H]^+^ 233.0720; found: 233.0725.

*3-(5-Bromo-1H-indole-3-yl) pyrrolidine-2,5-dione* (**3d**). White solid (84%), M.P.: 210–212 °C; ^1^H NMR (300 MHz, DMSO-d_6_) δ 11.32 (s, 1H), 11.26 (s, 1H), 7.64 (d, *J* = 1.1 Hz, 1H), 7.40 (d, *J* = 2.1 Hz, 1H), 7.35 (d, *J* = 8.6 Hz, 1H), 7.21 (dd, *J* = 8.6, 1.6 Hz, 1H), 4.36 (dd, *J* = 9.3, 5.5 Hz, 1H), 3.17 (dd, *J* = 18.0, 9.5 Hz, 1H), 2.80 (dd, *J* = 18.0, 5.5 Hz, 1H); ^13^C NMR (125 MHz, DMSO-d_6_) δ 180.2, 178.5, 135.6, 128.5, 125.3, 124.3, 121.4, 114.2, 111.8, 111.3, 39.1, 37.6; HRMS (ESI) calculated for C_12_H_10_BrO_2_N_2_ [M+ H]^+^ 292.99; found: 292.99.

*3-(2,5-dioxopyrrolidin-3-yl)-1H-indole-5-carbonitrile* (**3e**). Yellowish solid (56%), M.P.: 218–220 °C; ^1^H NMR (300 MHz, DMSO-d_6_) δ 11.57 (s, 1H), 11.30 (s, 1H), 8.45 (d, *J* = 1.2Hz, 1H), 7.94 (dd, *J* = 8.7, 1.8Hz, 1H), 7.42 (s, 2H), 4.39 (dd, *J* = 9.0, 5.4 Hz, 1H), 3.18 (dd, 1H), 2.79 (dd, 1H); ^13^C NMR (100 MHz, DMSO-d_6_) δ 177.4, 175.7, 139.0, 138.0, 124.6, 123.9, 118.6,115.0, 114.3, 112.0, 110.23, 36.9, 35.4; HRMS (ESI) calculated for C_13_H_9_N_3_O_2_ [M+ H]^+^ 240.0732; found: 240.0640.

*3-(5-Nitro-1H-indole-3-yl) pyrrolidine-2,5-dione* (**3f**). Yellow solid (60%), M.P.: 224–226 °C; ^1^H NMR (300 MHz, DMSO-d_6_) δ 11.59 (s, 1H), 11.32 (s, 1H), 8.47 (d, *J* = 1.6 Hz, 1H), 7.96 (dd, *J* = 9.0, 2.0 Hz, 1H), 7.44 (s, 2H), 4.41 (dd, *J* = 9.4, 5.5 Hz, 1H), 3.20 (dd, *J* = 18.1, 9.5 Hz, 1H), 2.81 (dd, *J* = 18.1, 5.5 Hz, 1H); ^13^C NMR (100 MHz, DMSO-d_6_) δ 177.6, 175.9, 139.1, 138.1, 124.8, 124.0115.2, 114.4, 112.1, 110.3, 37.0, 35.5; HRMS (ESI) calculated for C_12_H_9_O_4_N_3_Na [M+ Na]^+^ 282.0485; found: 282.0491.

*3-(1H-Indole-3-yl)-1-metHylpyrrolidine-2,5-dione* (**3g**). WHite solid (85%), M.P.: 170–172 °C; ^1^H NMR (300 MHz, DMSO-d_6_) δ 11.05 (s, 1H), 7.38 (d, *J* = 8.1 Hz, 2H), 7.33 (d, *J* = 2.3 Hz, 1H), 7.10 (t, *J* = 7.2 Hz, 1H), 6.99 (t, *J* = 7.1 Hz, 1H), 4.36 (dd, *J* = 9.3, 5.0 Hz, 1H), 3.23 (dd, *J* = 18.0, 9.4 Hz, 1H), 2.92 (s, 3H), 2.79 (dd, *J* = 18.0, 5.0 Hz, 1H); ^13^C NMR (75 MHz, CDCl_3_+ DMSO-d_6_) δ 177.4, 175.9, 175.6, 135.9, 124.8, 121.9, 120.9, 118.4, 117.3, 111.0, 109.7, 37.2, 35.6, 24.0; HRMS (ESI) calculated for C_13_ H_13_O_2_N_2_ [M+ H]^+^ 229.0971; found: 229.0973.

*3-(1H-indol-3-yl)-1-pHenylpyrrolidine-2,5-dione* (**3h**). WHite solid (78%), M.P.: 102–104 °C; ^1^H NMR (500 MHz, DMSO-d_6_) δ 11.10 (s, 1H), 7.55–7.49 (m, 3H), 7.46–7.41 (m, 2H), 7.40 (d, *J* = 8.1 Hz, 1H), 7.37–7.33 (m, 2H), 7.15–7.09 (m, 1H), 7.07–7.00 (m, 1H), 4.56 (dd, *J* = 9.5, 5.4 Hz, 1H), 3.41 (dd, *J* = 18.0, 9.6 Hz, 1H), 3.00 (dd, *J* = 18.0, 5.4 Hz, 1H); ^13^C NMR (100 MHz, DMSO-d_6_) δ 178.1, 176.2, 137. 0, 133.2, 129.4, 128.7, 127.6, 126.4, 124.2, 121.8, 119.4, 118.8, 112.2, 111.1, 38.4, 36.9; HRMS (ESI) calculated for C_18_ H_15_O_2_N_2_ [M+ H]^+^ 291.1128; found: 291.1128.

*1-Benzyl-3-(1H-Indole-3-yl) pyrrolidine-2,5-dione* (**3i**). WHite solid (88%), M.P.: 119–122 °C; ^1^H NMR (300 MHz, CDCl_3_) δ 8.18 (s, 1H), 7.45 (dd, *J* = 7.2, 2.2 Hz, 2H), 7.31 (dd, *J* = 5.4, 3.5 Hz, 3H), 7.24 (d, *J* = 4.5 Hz, 1H), 7.20 (d, *J* = 3.3 Hz, 1H), 7.16 (d, *J* = 7.3 Hz, 1H), 7.02 (d, *J* = 7.7 Hz, 1H), 6.99 (d, *J* = 2.7 Hz, 1H), 4.89–4.65 (m, 2H), 4.27 (dd, J = 9.5, 4.9 Hz, 1H), 3.24 (dd, J = 18.4, 9.5 Hz, 1H), 2.89 (dd, J = 18.4, 4.9 Hz, 1H); ^13^C NMR (75 MHz, CDCl_3_) δ 178.1, 176.2, 136.5, 135.7, 129.0, 128.7, 128.1, 125.4, 122.5, 122.4, 119.9, 118.4, 111.6, 111.2, 42.7, 38.2, 36.4; HRMS (ESI) calculated for C_19_ H_17_O_2_N_2_ [M+ H]^+^ 305.1284; found: 305.1289.

*1-Methyl-3-(5-nitro-1H-indole-3-yl)-pyrrolidine-2,5-dione* (**3j**). Yellow solid (64%), M.P.: 242–244 °C; ^1^H NMR (400 MHz, DMSO-d_6_) δ 11.82 (s, 1H), 8.56 (d, *J* = 2.1 Hz, 1H), 8.02 (dd, *J* = 9.0, 2.2 Hz, 1H), 7.63 (d, *J* = 2.1 Hz, 1H), 7.55 (d, *J* = 9.0 Hz, 1H), 4.55 (dd, *J* = 9.2, 5.4 Hz, 1H), 3.25 (dd, *J* = 17.9, 9.3 Hz, 1H), 3.02–2.91 (m, 1H), 2.91 (s, *J* = 4.6 Hz, 3H); ^13^C NMR (75 MHz, DMSO-d_6_) δ 177.9, 176.4, 140.5, 139.5, 126.8, 125.8, 116.7, 116.2, 113.6, 112.1, 37.0, 35.4, 24.6; HRMS (ESI) calculated for C_13_H_11_O_4_N_3_Na [M + Na]^+^ 296.0641; found: 296.0647.

*3-(5-MetHoxy-1H-indole-3-yl)-1-metHylpyrrolidine-2,5-dione* (**3k**). WHite solid (74%), M.P.: 138–140 °C; ^1^H NMR (300 MHz, DMSO-d_6_) δ 10.89 (s, 1H), 7.27 (d, *J* = 8.8 Hz, 2H), 6.87 (d, *J* = 2.0 Hz, 1H), 6.76 (dd, *J* = 8.8, 2.3 Hz, 1H), 4.33 (dd, *J* = 9.2, 5.0 Hz, 1H), 3.73 (s, 3H), 3.23 (dd, *J* = 17.9, 9.3 Hz, 1H), 2.92 (s, 3H), 2.79 (dd, *J* = 17.9, 4.9 Hz, 1H); 3H); ^13^C NMR (75 MHz, CDCl_3_ + DMSO-d6) δ 177.7, 175.9, 153.2, 131.4, 125.5, 122.5, 112.0, 111.3, 109.7, 99.8, 55.1, 37.4, 35.7; HRMS (ESI) calculated for C_14_ H_15_O_3_N_2_ [M + H]^+^ 259.1077; found: 259.1080.

*3-(5-Bromo-1H-indole-3-yl)-1-metHylpyrrolidine-2, 5-dione* (**3l**). WHite solid (70%), M.P.: 197–199 °C; ^1^H NMR (300 MHz, DMSO-d_6_) δ 10.62 (s, 1H), 7.49 (s, 1H), 7.22 (d, *J* = 8.6 Hz, 1H), 7.17–7.07 (m, 2H), 4.22 (dd, *J* = 9.3, 4.9 Hz, 1H), 3.20 (dd, *J* = 18.2, 9.4 Hz, 1H), 2.98 (s, 3H), 2.77 (dd, *J* = 18.2, 5.0 Hz, 1H);^13^C NMR (75 MHz, CDCl_3_ + DMSO-d_6_) δ 177.3, 175.6, 134.9, 127.0, 124.0, 123.2, 120.4, 112.8, 111.7, 109.6, 37.2, 35.7, 24.3; HRMS (ESI) calculated for C_13_ H_12_BrO_2_N_2_ [M + H]^+^ 307.0076; found: 307.0081.

*3-(5-Fluoro-1H-indole-3-yl)-1-metHylpyrrolidine-2, 5-dione* (**3m**). WHite solid (72%), M.P.: 200–202 °C; ^1^H NMR (300 MHz, DMSO-d_6_) δ 11.15 (s, 1H), 7.41 (d, J = 2.3 Hz, 1H), 7.37 (dd, *J* = 8.8, 4.6 Hz, 1H), 7.19 (dd, *J* = 10.0, 2.3 Hz, 1H), 7.00–6.89 (m, 1H), 4.35 (dd, *J* = 9.2, 5.2 Hz, 1H), 3.22 (dd, *J* = 17.9, 9.3 Hz, 1H), 2.91 (s, 2H), 2.87–2.75 (m, 1H); ^13^C NMR (125 MHz, DMSO-d_6_) δ 178.7, 177.0, 158.1 (d), 156.3, 133.5, 126.8, 126.8, 125.8, 113.1, 113.0, 111.4, 111.4, 110.1, 109.9, 103.9, 103.7, 37.8, 36.3, 25.0; HRMS (ESI) calculated for C_13_ H_12_FO_2_N_2_ [M + H]^+^ 247.0877; found: 247.0879.

*3-(5-Nitro-1H-indole-3-yl)-1-pHenylpyrrolidine-2,5-dione* (**3n**). Yellow solid (58%), M.P.: 106–108 °C;^1^H NMR (500 MHz, DMSO-d_6_) δ 11.87 (s, 1H), 8.62 (d, *J* = 2.1 Hz, 1H), 8.03 (dd, *J* = 9.0, 2.2 Hz, 1H), 7.74 (d, *J* = 2.1 Hz, 1H), 7.58 (d, *J* = 9.0 Hz, 1H), 7.52 (t, *J* = 7.7 Hz, 2H), 7.44 (t, *J* = 7.4 Hz, 1H), 7.34 (d, *J* = 7.4 Hz, 2H), 4.74 (dd, *J* = 9.4, 5.7 Hz, 1H), 3.45–3.41 (m, 1H), 3.15 (dd, *J* = 18.0, 5.7 Hz, 1H); ^13^C NMR (100 MHz,) δ 178.0, 176.2, 137.0, 133.2, 129.4, 128.7, 127.6, 126.4, 124.2, 121.8, 119.4, 118.8, 112.2, 111.1, 38.4, 36.9; MS–ESIMS: [M + H]^+^*m*/*z* 336.

*3-(5-MetHoxy-1H-indole-3-yl)-1-pHenylpyrrolidine-2,5-dione* (**3o**). White solid (78%), M.P.: 102–104 °C; ^1^H NMR (400 MHz, CDCl_3_) δ 8.13 (s, 1H), 7.53–7.47 (m, 2H), 7.44–7.39 (m, 1H), 7.38–7.34 (m, 2H), 7.28 (d, *J* = 8.9 Hz, 1H), 7.17 (d, *J* = 2.5 Hz, 1H), 6.98 (d, *J* = 2.3 Hz, 1H), 6.90 (dd, *J* = 8.8, 2.4 Hz, 1H), 4.48–4.43 (m, 1H), 3.83 (s, 3H), 3.45 (dd, J = 18.4, 9.6 Hz, 1H), 3.11 (dd, J = 18.4, 4.9 Hz, 1H); ^13^C NMR (100 MHz, CDCl_3_) δ 177.1, 175.5, 154.4, 132.0, 131.8, 129.2, 128.7, 126.5, 126.1, 122.8, 112.9, 112.5, 111.2, 100.5, 55.9, 38.4, 36.4; HRMS (ESI) calculated for C_19_ H_17_O_3_N_2_ [M + H]^+^ 321.1233; found: 321.1234.

*3-(5-Bromo-1H-indole-3-yl)-1-pHenylpyrrolidine-2, 5-dione* (**3p**). White solid (66%), M.P.: 212–214 °C; ^1^H NMR (300 MHz, DMSO-d_6_) δ 11.33 (s, 1H), 7.75 (s, 1H), 7.52 (t, *J* = 7.3 Hz, 2H), 7.44 (d, *J* = 7.2 Hz, 1H), 7.35 (dd, *J* = 13.4, 8.0 Hz, 2H), 7.23 (dd, *J* = 8.6, 1.5 Hz, 1H), 4.59 (dd, *J* = 9.3, 5.7 Hz, 1H), 3.05 (dd, *J* = 17.9, 5.6 Hz, 1H);^13^C NMR (75 MHz, DMSO-d_6_) δ 177.3, 175.5, 135.1, 132.6, 128.9, 128.3, 128.1, 127.1, 125.1, 123.9, 121.0, 113.7, 111.5, 110.5, 37.6, 36.1; HRMS (ESI) calculated for C_18_ H_14_BrO_2_N_2_ [M + H]^+^ 369.0233; found: 369.0246.

*3-(5-Fluoro-1H-indole-3-yl)-1-pHenylpyrrolidine-2, 5-dione* (**3q**). WHite solid (74%), M.P.: 130–132 °C; ^1^H NMR (400 MHz, DMSO-d_6_) δ 11.22 (s, 1H), 7.55–7.48 (m, 3H), 7.44 (d, *J* = 7.4 Hz, 1H), 7.39 (dd, *J* = 8.9, 4.6 Hz, 1H), 7.36–7.32 (m, 2H), 7.30 (dd, J = 10.1, 2.4 Hz, 1H), 6.97 (td, *J* = 9.2, 2.5 Hz, 1H), 4.56 (dd, *J* = 9.4, 5.6 Hz, 1H), 3.03 (dd, *J* = 18.0, 5.7 Hz, 1H); ^13^C NMR (126 MHz, DMSO-d6) δ 177.9, 176.1, 158.2 (d), 156.4, 133.6, 133.1, 129.4, 128.8, 127.6, 126.9, 126.9, 126.1, 113.2, 113.2, 111.4, 111.4, 110.2, 110.0, 103.9, 103.7, 38.2, 36.6; HRMS (ESI) calculated for C_18_H_14_FO_2_N_2_ [M + H]^+^ 309.1033; found: 309.1040.

*1-Benzyl-3-(5-nitro-1H-Indole-3-yl)-pyrrolidine-2,5-dione* (**3r**). Yellow solid (68%), M.P.: 182–184 °C; ^1^H NMR (300 MHz, DMSO-d_6_) δ 11.84 (s, 1H), 8.53 (d, *J* = 1.8 Hz, 1H), 8.01 (dd, *J* = 9.0, 2.0 Hz, 1H), 7.65 (d, *J* = 1.7 Hz, 1H), 7.55 (d, *J* = 9.0 Hz, 1H), 7.36–7.22 (m, 5H), 4.63 (s, 2H), 3.35–3.25 (m, 2H), 3.08 (dd, *J* = 18.1, 5.5 Hz, 1H); ^13^C NMR (75 MHz, DMSO-d_6_) δ 177.7, 176.2, 140.5, 139.5, 136.1, 128.5, 127.4, 127.1, 125.7, 116.8, 116.3, 113.5, 112.1, 41.6, 37.1, 35.2; HRMS (ESI) calculated for C_19_ H_15_O_4_N_3_Na [M + Na]^+^ 372.0954; found: 372.0969.

*1-Benzyl-3-(5-MetHoxy-1H-indole-3-yl)-pyrrolidine-2,5-dione* (**3s**). WHite solid (84%), M.P.: 139–141 °C; ^1^H NMR (500 MHz, CDCl_3_) δ 8.18 (s, 1H), 7.45 (d, *J* = 6.6 Hz, 2H), 7.31 (d, *J* = 7.3 Hz, 3H), 7.19 (d, *J* = 8.8 Hz, 1H), 6.94 (s, 1H), 6.83 (d, *J* = 8.6 Hz, 1H), 6.71 (s, 1H), 4.77 (q, *J* = 14.0 Hz, 2H), 4.24 (dd, *J* = 9.3, 4.9 Hz, 1H), 3.66 (s, 3H), 3.23 (dd, *J* = 18.4, 9.5 Hz, 1H), 2.91 (dd, *J* = 18.4, 4.8 Hz, 1H); ^13^C NMR (75 MHz, CDCl_3_) δ 178.0, 176.1, 154.3, 135.9, 131.7, 129.0, 128.7, 128.1, 126.0, 122.9, 112.9, 112.4, 111.0, 100.2, 55.7, 42.7, 38.3, 36.3; HRMS (ESI) calculated for C_20_H_19_O_3_N_2_ [M + H]^+^ 335.1390; found: 335.1398.

*1-Benzyl-3-(5-Bromo-1H-indole-3-yl)-pyrrolidine-2,5-dione* (**3t**). WHite solid (74%), M.P.: 167–169 °C; ^1^H NMR (300 MHz, CDCl_3_ + DMSO-d_6_) δ 10.42 (s, 1H), 7.37 (s, 1H), 7.30 (t, *J* = 7.4 Hz, 2H), 7.26 (s, 1H), 7.24–7.20 (m, 2H), 7.19 (s, 1H), 7.13 (dd, *J* = 8.6, 1.4 Hz, 1H), 7.04 (d, *J* = 2.2 Hz, 1H), 4.78–4.51 (m, 2H), 4.21 (dd, *J* = 9.4, 5.1 Hz, 1H), 3.19 (dd, *J* = 18.3, 9.5 Hz, 1H), 2.76 (d, *J* = 5.1 Hz, 1H); ^13^C NMR (75 MHz, CDCl_3_ + DMSO-d_6_) δ 177.2, 175.4, 135.3, 135.0, 128.2, 128.0, 127.4, 127.0, 124.3, 123.5, 120.4, 113.0, 112.0, 109.8, 42.0, 37.5, 35.8; HRMS (ESI) calculated for C_19_ H_15_BrO_2_N_2_Na [M + Na]^+^ 405.0209; found: 405.0220.

*1-Benzyl-3-(5-Fluoro-1H-indole-3-yl)-pyrrolidine-2,5-dione* (**3u**). WHite solid (70%), M.P.: 97–99 °C; ^1^H NMR (400 MHz, DMSO-d_6_) δ 11.16 (s, 1H), 7.42 (d, *J* = 2.4 Hz, 1H), 7.39 –7.28 (m, 6H), 7.09 (dd, *J* = 10.0, 2.3 Hz, 1H), 6.94 (td, *J* = 9.2, 2.5 Hz, 1H), 4.64 (s, 2H), 4.45 (dd, *J* = 9.3, 5.2 Hz, 1H), 3.30 (dd, *J* = 18.1, 9.4 Hz, 1H), 2.91 (dd, *J* = 18.1, 5.2 Hz, 1H); ^13^C NMR (100 MHz, DMSO-d_6_) δ 178.5, 176.8, 158.6 (d), 158.4, 156.0, 136.7, 133.6, 129.0, 128.1, 128.0, 126.7, 125.9, 113.2, 111.4, 110.2, 109.9, 103.9, 103.7, 42.1, 37.9, 36.2; HRMS (ESI) calculated for C_19_H_16_FO_2_N_2_[M + H]^+^ 323.1190; found: 323.1194.

*3-(1-methyl-2,5-dioxopyrrolidin-3-yl)-1H-indole-5-carbonitrile* (**3v**). Yellow solid (64%), M.P.: 238–240 °C; ^1^H NMR (400 MHz, DMSO-d_6_) δ 11.81 (s, 1H), 8.54 (d, *J* = 2.0 Hz, 1H), 8.0 (dd, 1H), 7.62 (d, *J* = 2.0 Hz, 1H), 7.53 (d, 1H), 4.54 (dd, 1H), 3.23 (dd, *J* = 17.6, 9.0 Hz, 1H), 3.0–2.90 (m, 1H), 2.90 (s, *J* = 4.3 Hz, 3H); ^13^C NMR (75 MHz, DMSO-d_6_) δ 177.7, 176.3, 140.4, 139.3, 126.7, 125.7, 118.6, 116.6, 116.1, 113.4, 112.0, 36.9, 35.3, 24.5; HRMS (ESI) calculated for C_14_H_11_N_3_O_2_Na [M + Na]^+^ 276.0732; found: 276.0633.

*1-metHyl-3-(1-metHyl-1H-indol-3-yl)-pyrrolidine-2,5-dione* (**3w**). WHite solid (84%), M.P.: 118–120 °C; ^1^H NMR (500 MHz, CDCl_3_) δ 7.41 (d, *J* = 8.0 Hz, 1H), 7.32 (d, *J* = 8.2 Hz, 1H), 7.25 (t, *J* = 7.3 Hz, 1H), 7.12 (t, *J* = 7.5 Hz, 1H), 7.03 (s, 1H), 4.29 (dd, *J* = 9.4, 4.9 Hz, 1H), 3.75 (s, 3H), 3.27 (dd, *J* = 18.3, 9.5 Hz, 1H), 3.10 (s, 3H), 2.92 (dd, *J* = 18.3, 4.9 Hz, 1H); ^13^C NMR (125 MHz, CDCl_3_) δ 178.4, 176.6, 137.4, 126.8, 126.2, 122.3, 119.7, 118.6, 110.0, 109.8, 38.2, 36.7, 32.8, 25.2; HRMS (ESI) calculated for C_14_H_15_O_2_N_2_ [M + H]^+^ 243.1128; found: 243.1131.

*1-Benzyl-3-(1-Benzyl-5-MetHoxy-1H-indole-3-yl) pyrrolidine-2,5-dione* (**3x**). White solid (90%), M.P.: 147–149 °C; ^1^H NMR (300 MHz, CDCl_3_) δ 7.48–7.39 (m, 2H), 7.28 (t, *J* = 6.6 Hz, 6H), 7.17–7.05 (m, 3H), 6.99 (s, 1H), 6.82 (dd, *J* = 8.9, 2.2 Hz, 1H), 6.73 (d, *J* = 2.0 Hz, 1H), 5.20 (s, 2H), 4.88–4.60 (m, 2H), 4.26 (dd, *J* = 9.4, 4.9 Hz, 1H), 3.65 (s, 3H), 3.25 (dd, *J* = 18.3, 9.5 Hz, 1H), 2.93 (dd, *J* = 18.3, 4.9 Hz, 1H); ^13^C NMR (75 MHz, CDCl_3_) δ 178.04, 176.16, 154.29, 135.86, 131.66, 129.00, 128.75, 128.0, 126.0, 123.0, 112.9, 112.4, 111.0, 100.2, 55.7, 42.7, 38.3, 36.3; HRMS (ESI) calculated for C_27_H_25_O_3_N_2_ [M + H]^+^ 425.1859; found: 425.1868.

### Appendix A.3. Biology

#### Cell Cultures, Maintenance and Anti-Proliferative Evaluation

The cell linesHT-29, Hepg-2 and A549 (colorectal, liver and lung cancer cells) used in this study was procured from American Type Culture Collection (ATCC), USA. Thesynthesized test compounds were evaluated for theirin vitro anti-proliferative activity in thesethree different human cancer cell lines. A protocol of 48 hcontinuous drug exposure was used, and an SRB cell proliferation assay was used to estimate cell viability or growth. All the cell lines were grown in Dulbecco’s modified Eagle’s medium (containing 10% FBS in a humidified atmosphere of 5% CO_2_ at 37 °C). Cells were trypsinized when sub-confluent from T25 flasks/60 mm dishes and seeded in 96-well plates in 100 μL aliquots at plating densities depending on the doubling time of individual cell lines. The micro-titer plates were incubated at 37 °C, 5% CO_2_, 95% air, and 100% relative humidity for 24 hprior to the addition of experimental drugs and were incubated for 48 h with different doses (0.01, 0.1, 1, 10, 100 μM) of the prepared derivatives. After incubation at 37 °C for 48 H, the cell monolayers were fixed by the addition of 10% (wt/vol) cold trichloroacetic acid and incubated at 4 °C for 1 hand were then stained witH0.057% SRB dissolved in 1% acetic acid for 30 min at room temperature. Unbound SRB was washedwith 1% acetic acid.

### Appendix A.4. Molecular Docking Study

#### Appendix A.4.1. Protein Preparation and Grid Generation

The 3D coordinates of the CDK2 catalytic core in complex withStaurosporine were taken from theRCSB Protein Databank (PDB code: 1AQ1) The PDB protein-ligand structures were processed withthe Protein Preparation Wizard in theSchrodinger suite. The protein structure integrity was checked and adjusted, and missing residues and loop segments near the active site were added using Prime. A 3D box was generated around each ligand to enclose the entire vicinity of active site. The receptor grid for each target was prepared withthehelp of OPLS_2005 force field. The grid center was set to be the centroid of the co-crystallized ligand, and the cubic grid Had a size of 20 Å.

#### Appendix A.4.2. Ligand Preparation

The 2D ligand structures were prepared using Chem-BioDraw Ultra 12.0, and the 3D structures were generated by Schrodinger suite. Schrodinger’sLigPrep program was used to generate different conformations of ligands.

#### Appendix A.4.3. Molecular Docking

Molecular docking studies were performed by using a GLIDE docking module of Schrodinger suite. For the validation of docking protocol, the co-crystalized ligand (STU in 1AQ1) was subjected to re-docking into the CDK2 (PDB code: 1AQ1) using GLIDE. The bound and docked conformations of STU (RMSD 0.8170 Å) showed similar interactions and binding pose at their respective binding sites. Finally, prepared ligands were docked into the generated receptor grids using Glide SP docking precision. The results were analyzed on the basis of the GLIDE docking score and molecular recognition interactions. All the 3D figures were obtained using Schrödinger Suite 2014-3.
